# Palatability Enhancement Potential of *Hermetia illucens* Larvae Protein Hydrolysate in *Litopenaeus vannamei* Diets

**DOI:** 10.3390/molecules26061582

**Published:** 2021-03-13

**Authors:** David Terrey, Jack James, Ivan Tankovski, Monika Dalim, Michel van Spankeren, Arpita Chakraborty, Eric Schmitt, Aman Paul

**Affiliations:** 1 Pontus Research Limited, Hirwaun CF44 9UP, UK; det@pontusresearch.com (D.T.); jmj@pontusaqua.com (J.J.); ivt@pontusresearch.com (I.T.); 2 Protix B.V., 5107 NC Dongen, The Netherlands; monika.dalim@protix.eu (M.D.); Michel.vanspankeren@protix.eu (M.v.S.); arpita.chakraborty@protix.eu (A.C.); eric.schmitt@protix.eu (E.S.)

**Keywords:** insect, protein hydrolysate, free amino acids, short chain peptides, *Litopenaeus vannamei*, palatability

## Abstract

Marine feed ingredients derived from cephalopods (e.g., squid) and crustaceans (e.g., krill) are commercially used to improve the palatability of shrimp diets. Increase in global demand for shrimps has resulted in overfishing of these marine organisms and is a matter of concern. Insect protein hydrolysate could be a sustainable alternative for the possible replacement of these marine feed ingredients. During this study, four formulations: diet A (control: not containing any palatability enhancer), diet B (containing squid meal and krill oil), diet C (containing 1% insect protein hydrolysate), and diet D (containing 2% insect protein hydrolysate) were tested for (1) time required by first subject to begin feeding (time to strike) and (2) palatability in *Litopenaeus vannamei*. Additionally, the chemical composition of all four diet formulations was also analyzed. Results indicate that all diets had similar crude composition. The major essential amino acids in all diets were leucine and lysine, whereas eicosapentaenoic acid was the major omega-3 fatty acid in all diets. There were no significant differences between the mean time to strike for all the tested formulations. Palatability of tested formulations was found in the following order: diet D > diet C > diet B = diet A (*p* < 0.05), indicating that addition of squid meal and krill oil has no effect on palatability in comparison to control, whereas inclusion of insect protein hydrolysates significantly improves the palatability of formulations. Palatability enhancement potential of insect protein hydrolysate could be attributed to the high free amino acid content and water solubility in comparison to squid meal.

## 1. Introduction

Globally, around 15 million tons of whole fish is used for the production of fishmeal [1]. These figures have raised serious concerns related to the overfishing of pelagic fishes that are used for the production of fishmeal [2]. Hence, researchers and industries are currently evaluating the potential of plant proteins (mainly soymeal) for the partial substitution of fishmeal in aquaculture feed formulations [3]. However, replacement of fishmeal with plant proteins has a direct impact on the palatability of diets. Commercially important aquaculture species, such as Pacific white shrimp (*Litopenaeus vannamei*), less readily consume diets containing plant proteins due to low attractability and palatability [4]. In order to overcome this challenge, companies are currently including other marine feed ingredients (e.g., squid and krill derived ingredients) for palatability improvement. These ingredients contain molecules such as short chain peptides, free amino acids, etc., that are recognized by the sensory system of crustaceans for the location and oral ingestion of feed [3]. Increase in demand and overfishing of these marine organisms is again a matter of concern [5,6]. For squids, researchers believe that overfishing combined with deteriorating environmental conditions could push the population below critical levels [7]. Insect derived feed ingredients, such as insect proteins, are gaining popularity due to (a) nutritional benefits and (b) circularity in production chains [8,9,10]. Insect protein derivatives could present interesting candidature for the replacement of squid and krill derived ingredients to improve palatability of shrimp diets.

Companies are developing insect derived protein hydrolysates for positioning in animal feed markets. Some of these products have all the proteins in the form of short chain peptides (<1000 da) that have strong health promoting potential [11] and contain high levels of free amino acids, which could potentially improve the palatability of shrimp diets. However, to the best of the authors knowledge, there are no studies that have evaluated the potential of insect protein hydrolysate to replace squid and krill derived ingredients for the purpose of *L. vannamei* diet palatability enhancement.

The current study reports the evaluation of palatability aspects of insect protein hydrolysate by replacement of squid and krill derived ingredients in *L. vannamei* diets. During this study, we analyzed (a) the proximate composition, fatty acid profile, and amino acid composition, (b) the palatability, and (c) the time to strike for diets containing insect protein hydrolysates in comparison to diets containing squid and krill derived ingredients.

## 2. Results

### 2.1. Feed Composition

Proximate composition of test diets is mentioned in Table 1, indicating that all four tested diets had similar protein, fat, ash, and energy contents. Amino acid composition of four diets are mentioned in Table 2. Glutamic acid and aspartic acid were the major amino acids present in all four diets, whereas leucine and lysine were the major essential amino acids present in all four diets.

Fatty acid composition of four diets are mentioned in Table 3. Palmitic acid (C 16:0) and oleic acid (C 18:1) were the major fatty acids present in all four diets. Eicosapentaenoic acid (C 20:5n3) was the omega-3 fatty acid that was found in major concentrations in all diets. Diet A (control diet) was analyzed to contain highest levels of eicosapentaenoic acid and docosahexaenoic acid (C 22:6n3) amongst the four diets. On the other hand, diet B (containing squid meal and krill oil) was analyzed with lowest levels of these omega-3 fatty acids.

### 2.2. Assessment of Time to Strike

**Figure 1:**
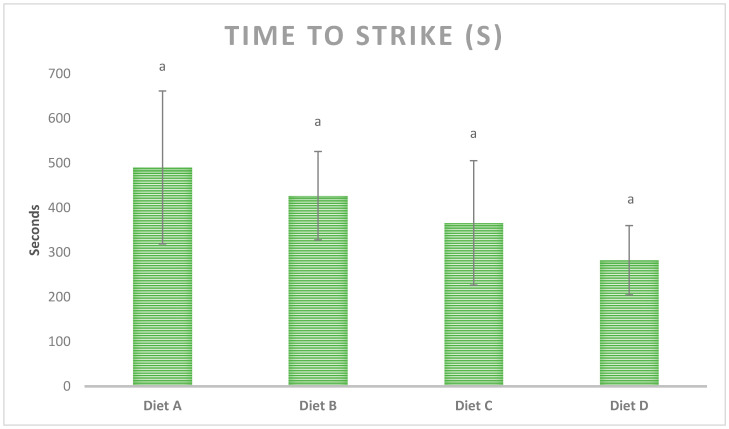
Time taken by the first shrimp to begin feeding (time to strike) for diets used in this study. Data is presented as mean ± standard deviation (*n* = 5). Different letters in the same row indicate significant differences (*p* < 0.05). Diet A: containing no palatability enhancers (control); diet B: containing squid meal and krill oil; diet C: containing 1% insect protein hydrolysate; diet D: contain 2% insect protein hydrolysate.

The time to strike for all four diets is indicated in Figure 1. The mean time to strike values were in the following order: diet A > diet B > diet C > diet D. It gives an indication that inclusion of squid meal and krill oil and insect protein hydrolysate decreased the time to strike. However, there were no significant differences between the time to strike of various diets (*p* < 0.05).

### 2.3. Assessment of Palatability

**Figure 2:**
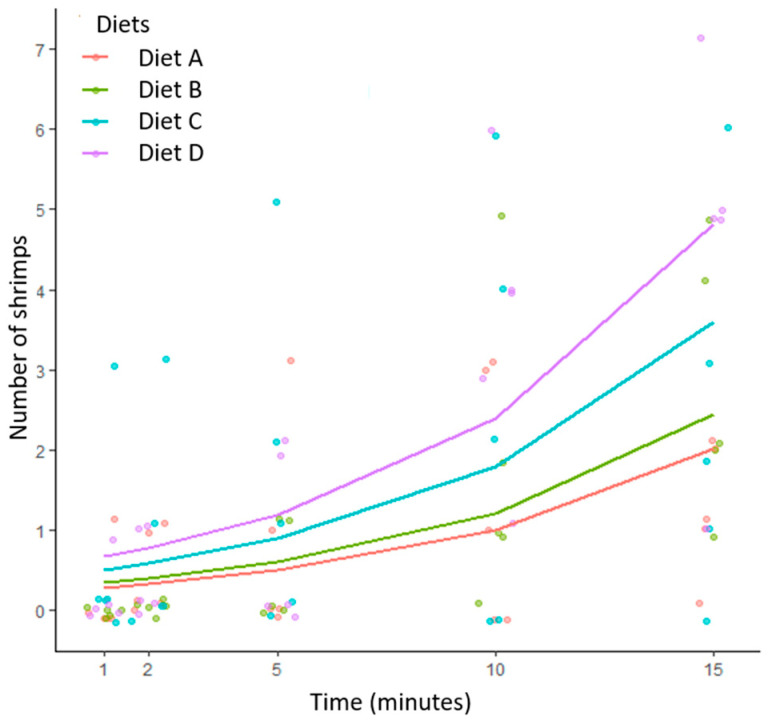
Jittered count observations at each time interval with the predicted performance fit for all four diets over time. Diet A: containing no palatability enhancers (control); diet B: containing squid meal and krill oil; diet C: containing 1% insect protein hydrolysate; diet D: contain 2% insect protein hydrolysate.

The number of shrimps on all four diets after 1, 2, 5, 10, and 15 min (palatability) is indicated in Figure 2. The diets were in the following order of palatability: diet D > diet C > diet B = diet A (*p* < 0.05). It appears that adding squid meal and krill oil has no significant effect on palatability improvement, whereas inclusion of insect protein hydrolysate significantly improves the palatability of shrimp diets.

## 3. Discussion

The Pacific white shrimp is one of the most widely reared crustacean species for human food [12]. Shrimps have a highly developed chemosensory system [13]. In crustaceans, the following chemoreceptors play a key role in sensory perception of feed: (a) antennular chemoreceptors, by searching and orientation of chemical stimulus; (b) leg chemoreceptors, by stimulating local grasping reflexes; (c) mouth chemoreceptors, by having control over decision to ingest food [14,15]. Molecules that can interact with these crustacea chemoreceptors to generate positive response are known to effectively enhance palatability. These molecules include free amino acids, short chain peptides, nucleotides, etc. [3,14].

Squid meal is currently added in shrimp diets to enhance palatability [16], and contains up to 1% to 3% free amino acids on a dry matter basis [3,17]. Krill oil is often used in shrimp diets to enhance palatability in combination with marine proteins [18]. Insect protein hydrolysate contains > 22% free amino acids on a dry matter basis (considering 45% proteins are present as free amino acids, Table 4) and the rest of the proteins are present as short chain peptides. Additionally, insect protein hydrolysate used in this study is 100% water soluble (Section 4), suggesting that the inclusion of this hydrolysate could be beneficial for the purpose of palatability enhancement in shrimp diets.

The first part of this study involved chemical characterization of the four diets used for palatability enhancement. Recommended minimum dietary protein content of *L. vannamei* diets is between 300 to 360 g/kg depending on the growth stage and environmental conditions [19,20], whereas recommended minimal calorific values of *L. vannamei* diets are 12.5 MJ/kg, respectively [20]. During this study, all the four diets were analyzed with protein contents between 39% to 40% and calorific value of 20 MJ/kg, indicating that all the tested diets were able to supply sufficient quantities of proteins and energy. Regarding the essential amino acids, *L. vannamei* diets should supply minimum 14.3, 7.1, 8.8, 15, 15.4, 6.2, 12.4, 10.5, and 11.1 g/kg of arginine, histidine, isoleucine, leucine, lysine, methionine, phenylalanine, threonine, and valine, respectively [20]. Fatty acid requirements for shrimps are dependent on both species and life stage. Researchers have indicated that *L. vannamei* adults require at least 0.5% of dietary omega-3 fatty acids for optimal growth [21]. Again, all the four diets used in this study supply these essential amino acids and omega-3 fatty acids in sufficient quantities (Table 2 and Table 3). High polar lipid krill oil is commonly used in aquaculture feeds [22]. This oil contains 16.4% and 9.5% of eicosapentaenoic acid and docosahexaenoic acid, respectively [23], whereas oil from fishes like anchovies contains up to 26.0% and 26.5% eicosapentaenoic acid and docosahexaenoic acid, respectively [24]. This could possibly explain the reason behind lower amounts of eicosapentaenoic acid and docosahexaenoic acid in diet B where fish oil was partially supplemented by krill oil. Comparing the nutritional value of four diets is not the main objective of this study; however, it does provide an indication that all the diets were nutritionally sufficient.

The main goal of the study was to address the palatability enhancement potential of insect protein hydrolysate in shrimp diets. The inclusion of squid meal and krill oil reduced the mean time to strike in comparison to the control diet, whereas insect protein hydrolysate (both at 1% and 2%) reduced the mean time to strike even further when compared to the combination of squid meal and krill oil (*p* > 0.05). Reduction in the mean time to strike could be related to the ability of target molecules to be sensed by antennular of shrimps and ability (or speed) of shrimps to reach the feed [2,3,14]. In order to maximize antennular sensing, a palatability enhancer should be highly water soluble. Squid meal has a water solubility of 11% [25], whereas insect protein hydrolysate has water solubility close to 100% (Section 4). A palatability enhancer should also contain high levels of free amino acids. Squid meals contain up to 3% free amino acids, whereas insect protein hydrolysate contains up to 22% free amino acids. Both these features provide a clear advantage to insect protein hydrolysate. Additionally, the polar lipid content of krill oil could also be a contributing factor. Krill oil contains up to 35% polar lipids [26]. These fatty molecules are known to effectively stabilize protein based emulsions [27] and could present at least some resistance for the free amino acids to leaching.

The time to strike may have also been affected by the shrimp’s ability to reach the feed. *L. vannamei* can swim up to four to five times their body length per second [28]; however, decision time and obstructions will further reduce the speed. Hence, it appears that even if compositional characteristics of insect protein hydrolysate enables effective dispersion and sensing by antennular of shrimps, locomotor limitation may affect the response times. Therefore, the mean time to strike decreased by addition of insect protein hydrolysate, but the reduction was not enough to observe significant differences.

The ability of insect protein hydrolysate to improve the palatability of shrimp diets could be attributed to high levels of free amino acids. A previous study has shown that increasing free amino acids levels in diets has a dose dependent effect on palatability improvement for some crustacean species. In the same study, it was found that free arginine, glutamic acid, and phenylalanine are strong palatability enhancers for crustaceans like *Gnathophausia ingens* and *Pagurus hirsutiusculus* [29]. According to the literature, squid meal contains up to 0.7%, 0.1%, and 0% of free arginine, glutamic acid, and phenylalanine, respectively [3,17]. From Table 5, it is evident that insect protein hydrolysate contain much higher levels of all these free amino acids in comparison to squid meal. Perhaps, this also explains the reason why most studies have considered using 3% squid meal inclusion levels for palatability assessment [3,30]. Results of the current study indicate that insect protein hydrolysate could enhance the palatability of shrimp diets even at 1% inclusion levels.

A feeding response trial on *L. vannamei* has indicated the efficacy of peptide mix (where the majority of peptides are <2500 da) on the improvement of growth performance and immunity [31]. The insect protein hydrolysate used in this study has all the proteins <1000 da; whereas some researchers have also reported the immune response modulation activity of this hydrolysate during in vitro trials [11]. In the future, it could be interesting to study the growth and health response of shrimps when fed with diets containing insect protein hydrolysate. Apart from the sustainability aspect and strong palatability enhancement potential indicated in this study, the ability of this product to stimulate growth and health of shrimps will add to the potential benefits.

## 4. Materials and Methods

*Hermetia illucens* larvae protein hydrolysate was supplied by Protix B.V. (Dongen, The Netherlands). According to the supplier, this insect protein hydrolysate was 100% water soluble. The composition of insect protein hydrolysate used in this study is indicated in Table 4, whereas the free amino acid profile of insect protein hydrolysate used in this study is indicated in Table 5.

### 4.1. Feed Preparation

The feeds were formulated by Pontus Research Limited (Aberdare, UK). Ingredient mixing and pellet production were done by Sparos (Olhao, Portugal). In total, four diets (a control diet, a diet containing squid meal and krill oil, two diets contaning 1% and 2% insect protein hydrolysate, respectively) were prepared. The feed formulations used in this study are indicated in Table 6.

### 4.2. Feed Composition Analysis

Compositional analysis of diets A, B, C, and D was performed by Nutrilab BV (Giessen, The Netherlands). List of analyses and respective methods used during this study are indicated in Table 7. All the analyses were performed in triplicates.

### 4.3. Experimental Animals and Setup

The feeding trial was conducted at Pontus Research Limited. Animals were housed in a recirculating aquaculture system (RAS). Total of 11 holding tanks were used in the study (one test tank and 10 buffer tanks for purpose of cleaning or repairing). The incoming water was carbon filtered and filtered via fluidized sand tower. Chemical quality of water in tanks was measured biweekly, to adhere with following parameters: ammonia < 0.03 mg/mL, nitrogen dioxide < 0.6 mg/mL, and nitrates < 75 mg/L using HANNA Multiparameter Bench Photometer (Leighton Buzard, UK). Temperature was controlled using inline heaters in holding tanks (28 ± 1 °C). Oxygen levels (>80% saturation), pH (8.00 ± 0.25), and salinity (15 parts per thousands) were also monitored on a daily basis. Light/dark cycle of 12/12 h was maintained during the study.

**Figure 3:**
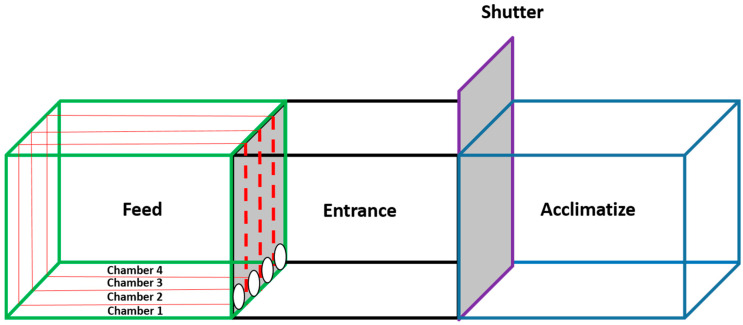
Pictorial representation of test setup used for shrimp palatability testing.

Test tank setup had a capacity of 140 L and consisted of a glass tank of 90 × 30 × 30 cm (length × width × height). The tank had an acclimatization chamber at one end and four feeding chambers at the other end (Figure 3. A movable glass shutter separated the acclimatization and feeding chambers. Each feeding chamber had an opening to allow free access of shrimps to the feed in the chamber. The tank was set up in a closed room, with continuous levels of diffused light throughout the trial period. Water was circulated and maintained with an air driven sponge filter throughout the testing period. The temperature was maintained with the use of submersible heaters (28 ± 1 °C).

In total, 220 adult (initial weight 30 to 35 g) Pacific white shrimp were used for the experiment. All shrimps used in the trial were domesticated and had high health, derived from a UK based supplier. Animals were transported at the post-larval stage and grown in holding tanks using commercial shrimp feeds until they reached required sizes necessary for experimentation. Animal welfare was ensured by (a) minimal handling of animals, (b) maintaining water quality parameters, and (c) daily observation of animal health and wellbeing. Sick animals (if any) were immediately removed and euthanized humanely. 

### 4.4. Assessment of Time to Strike

Shrimps (10 per test) were placed in acclimatization chamber for 30 min before the start of testing. Following this, 2 g of a single feed was randomly placed in one of the four chambers in the feed section of test setup. The feed was then left for 10 min to allow for dissipation of attractants into the water. Finally, the shutter between the acclimatization chamber and entrance chamber was lifted. The time taken by the first shrimp to begin feeding was recorded (which is referred to as time to strike). The experiment was repeated five times for each feed. Between repetitions, the tank was completely drained and cleaned to ensure no smell from previous repetitions remained.

### 4.5. Assessment of Palatability

Shrimps (10 per test) were placed in the acclimatization chamber 30 min before the start of testing. Following this, 2 g of each feed was randomly allocated to four chambers of the feed section of test setup. The feed was placed in the appropriate chamber then left for 10 min to allow for dissipation of attractants into the water. Finally, the shutter between acclimatization chamber and entrance chamber was lifted. The number of shrimps in all four chambers of feed section were recorded after 1, 2, 5, 10, and 15 min (which is referred as palatability). The experiment was repeated five times. Between repetitions, the tank was completely drained and cleaned to ensure no smell from previous repetitions remained.

### 4.6. Statistical Analysis

Analysis of time to strike and palatability data was realized using R statistical software (version 4.0.2). Palatability data was modelled using a quasi-Poisson distribution, which allows the dispersion parameters to differ from the mean in the model. Squid meal and krill oil inclusion was accounted for as a dummy variable, and insect protein hydrolysate inclusion was accounted for as a continuous variable, with value being the level of inclusion. As an output of this model, we obtained a parameter estimate for the effect of insect protein hydrolysate inclusion on shrimp counts. Time to strike data was analyzed using ANOVA coupled with Shapiro—Wilk test for normality and Levene’s test for homogeneity of variance confirmation. If the normality and homogeneity of variance was confirmed, then ANOVA was accepted and post-hoc pairwise Tukey’s test was used to estimate *p*-values. If the normality and homogeneity of variance was not confirmed, a Kruskall—Wallace nonparametric test was carried out on the data with a post-hoc pairwise Conover—Iman analysis to estimate *p*-values. The differences were considered significant if *p* < 0.05.

## Figures and Tables

**Table 1:** Proximate composition of four diets used in this study.

Component	Diet A (Control: No Palatability Enhancers Added)	Diet B (Containing Squid Meal and Krill Oil)	Diet C (Containing 1% Insect Protein Hydrolysate)	Diet D (Containing 2% Insect Protein Hydrolysate)
Moisture (g/kg)	4.3 ± 0.0	5.5 ± 0.0	4.3 ± 0.0	4.4 ± 0.0
Crude protein (g/kg)	39.7 ± 0.2	39.3 ± 0.3	39.0 ± 0.2	39.4 ± 0.3
Fat (g/kg)	9.0 ± 0.2	9.0 ± 0.1	9.1 ± 0.0	9.0 ± 0.1
Ash (g/kg)	7.9 ± 0.1	7.5 ± 0.1	7.8 ± 0.1	7.9 ± 0.0
Calorific value (MJ/kg)	20.3 ± 0.0	20.3 ± 0.0	20.3 ± 0.0	20.3 ± 0.0

Data are presented as mean ± standard deviation (*n* = 3).

**Table 2:** Total amino acid composition of four diets used in the study.

Amino Acids (g/kg Feed)	Diet A (Control: No Palatability Enhancers Added)	Diet B (Containing Squid Meal and Krill Oil)	Diet C (Containing 1% Insect Protein Hydrolysate)	Diet D (Containing 2% Insect Protein Hydrolysate)
Alanine	22.0 ± 0.8	22.0 ± 1.0	23.2 ± 2.0	22.0 ± 0.5
Arginine	24.0 ± 0.9	24.0 ± 1.0	24.1 ± 0.5	22.8 ± 1.5
Aspartic acid	36.1 ± 0.3	35.0 ± 0.0	36.4 ± 0.9	36.0 ± 0.8
Cystine	5.0 ± 0.6	4.8 ± 0.5	5.1 ± 0.5	5.0 ± 0.1
Glutamic acid	73.0 ± 0.2	69.2 ± 0.1	69.9 ± 0.1	68.0 ± 0.9
Glycine	25.8 ± 0.5	26.3 ± 0.3	25.9 ± 0.3	25.8 ± 0.3
Histidine	9.1 ± 0.3	9.4 ± 0.4	9.1 ± 0.4	9.3 ± 0.5
Isoleucine	15.8 ± 0.4	16.2 ± 0.6	16.4 ± 0.4	15.9 ± 0.4
Leucine	28.1 ± 0.4	28.3 ± 0.6	28.4 ± 0.3	27.7 ± 0.2
Lysine	25.3 ± 0.1	26.4 ± 0.2	25.5 ± 0.0	25.0 ± 0.0
Methionine	8.6 ± 0.7	8.7 ± 0.4	9.0 ± 0.3	8.7 ± 0.3
Phenylalanine	18.8 ± 3.2	16.8 ± 0.6	17.0 ± 0.3	16.7 ± 0.6
Proline	22.4 ± 1.3	22.1 ± 0.7	20.6 ± 0.3	21.8 ± 0.6
Serine	18.3 ± 0.1	18.0 ± 0.3	17.5 ± 0.3	18.4 ± 0.6
Threonine	15.4 ± 0.2	15.4 ± 0.2	14.1 ± 2.0	15.4 ± 0.5
Tryptophan	0.4 ± 0.0	0.4 ± 0.0	0.4 ± 0.0	0.3 ± 0.0
Tyrosine	12.3 ± 0.6	13.0 ± 0.6	12.2 ± 0.5	12.7 ± 0.9
Valine	18.5 ± 0.8	18.4 ± 0.7	18.9 ± 0.6	18.7 ± 0.9

Data are presented as mean ± standard deviation (*n* = 3).

**Table 3:** Fatty acid composition of four diets used in the study.

Fatty Acids (g/kg Fat)	Diet A (Control: No Palatability Enhancers Added)	Diet B (Containing Squid Meal and Krill Oil)	Diet C (Containing 1% Insect Protein Hydrolysate)	Diet D (Containing 2% Insect Protein Hydrolysate)
Lauric acid	4.0 ± 0.6	2.0 ± 1.5	1.0 ± 0.0	6.0 ± 0.0
Myristic acid	74.0 ± 4.6	87.0 ± 9.8	74.0 ± 1.5	73.0 ± 3.8
Pentadecanoic acid	5.0 ± 0.0	5.0 ± 0.6	5.0 ± 0.0	5.0 ± 0.0
Palmitic acid	212.0 ± 9.8	229 ± 23.8	217.0 ± 6.1	210.0 ± 10.8
Palmitoleic acid	69.0 ± 3.5	82.0 ± 7.2	70.0 ± 1.5	69.0 ± 2.9
Heptadecanoic acid	4.0 ± 0.6	4.0 ± 0.6	5.0 ± 0.6	5.0 ± 0.6
Stearic acid	43.0 ± 1.5	43.0 ± 4.2	46.0 ± 1.5	44.0 ± 3.6
Oleic acid	109.0 ± 4.4	125.0 ± 10.0	113.0 ± 2.6	114.0 ± 8.5
Linoleic acid	109.0 ± 1.0	99.0 ± 7.2	107.0 ± 0.6	110.0 ± 1.5
α-Linolenic acid	17.0 ± 0.6	15.0 ± 1.5	17.0 ± 0.6	18.0 ± 0.6
Arachidic acid	3.0 ± 0.0	2.0 ± 0.6	3.0 ± 0.0	3.0 ± 0.6
Eicosadienoic acid	4.0 ± 0.0	3.0 ± 1.2	4.0 ± 0.0	4.0 ± 0.0
Arachidonic acid	10 ± 0.6	7.0 ± 2.1	9.0 ± 0.6	9.0 ± 1.5
Eicosapentaenoic acid	123.0 ± 13.1	90.0 ± 29.5	116.0 ± 7.0	115.0 ± 21.7
Behenic acid	2.0 ± 0.0	2.0 ± 0.0	2.0 ± 0.0	2.0 ± 0.6
Erucic acid	6.0 ± 0.0	7.0 ± 0.6	6.0 ± 0.6	6.0 ± 0.6
Docosabexaenoic acid	90.0 ± 12.8	76.0 ± 26.2	84.0 ± 7.0	82.0 ± 21.8
Lignoceric acid	1.0 ± 0.6	10.0 ± 0.6	2.0 ± 0.0	1.0 ± 0.6
Nervonic acid	11.0 ± 0.0	10.0 ± 0.6	12.0 ± 0.6	11.0 ± 0.0

Data are presented as mean ± standard deviation (*n* = 3).

**Table 4:** Chemical composition of insect protein hydrolysate (as declared by supplier).

Component	Insect Protein Hydrolysate ^1^
Moisture (g/kg)	55
Crude protein (g/kg)	455
Crude fat (g/kg)	35
Proteins < 1000 da (% of total proteins)	>98
Free amino acids (% of total proteins)	45

^1^ Supplier is selling this product under the brand name *ProteinAX_pro_*.

**Table 5:** Free amino acid profile of insect protein hydrolysate (as declared by supplier).

Component (g/kg)	Free Amino Acids
Alanine	32
Arginine	20
Aspartic acid	9
Cystine	2
Glutamic acid	16
Glycine	10
Histidine	11
Isoleucine	6
Leucine	10
Lysine	14
Methionine	1
Phenylalanine	7
Proline	20
Serine	8
Threonine	10
Tryptophan	4
Tyrosine	15
Valine	13

**Table 6:** Ingredient composition of the basal and experimental diets.

Component	Diet A (Control: No Palatability Enhancers Added)	Diet B (Containing Squid Meal and Krill Oil)	Diet C (Containing 1% Insect Protein Hydrolysate)	Diet D (Containing 2% Insect Protein Hydrolysate)
Fish Meal LT70 (G/Kg)	300.0	300.0	300.0	300.0
Soymeal 48 (G/Kg)	250.0	193.0	240.3	230.7
Wheat	389.6	417.8	389.6	389.8
Squid Meal 83	0.0	30.0	0.0	0.00
Insect Protein Hydrolysate	0.0	0.0	10.0	20.0
Fish Oil	46.0	34.7	45.9	45.7
Krill Oil	0.0	10.0	0.0	0.0
Premix *	10.0	10.0	10.0	10.0
Calcium Carbonate	3.6	3.5	3.8	3.8
MCP	0.8	1.0	0.4	0.0
Total	1000.0	1000.0	1000.0	1000.0

* Composition of Premix (expressed as per kg of premix): Vit A (Retinol acetate)—2,000,000 U.I., Vit D3 (Cholecalciferol)—200,000 U.I., Vit K3 (Menadione sodium bisulfite)—2500 mg, Vit B1 (Thiamine HCl)—3000 mg, Vit B2 (Riboflavine)—3000 mg, Vit B3 (Niacin / Nicotinic acid)—20,000 mg, Vit B5 (Calcium panthotenate)—10,000 mg, Vit B6 (Pyridoxine HCL)—2000 mg, Vit B9 (Folic acid)—1500 mg/kg, Vit B12 (Cyanocobalamin)-10 mg, Vit H (Biotin)—300 mg, Vit E (α-Tocopherol acetate)—10,000 mg, Vit C (L-ascorbic acid monophosphate)—100,000 mg, Inositol—50000 mg, Betaine—50,000 mg, Choline chlorine-100,000 mg, Copper sulfate (5H_2_O)—900 mg, Ferric sulfate—600 mg, Potassium Iodide—50 mg, Manganese oxide—960 mg, Sodium selenite—10 mg, Zinc sulfate—750 mg, Calcium—60,000 mg, and Chlorine—25,000 mg.

**Table 7:** Compositional analyses of feed performed during this study.

Analysis	Method
Moisture content	Regulation (EC) 152/2009
Crude protein—Dumas N*6.25	NEN-EN-ISO 16634
Crude fat (after pre-extraction and hydrolysis)	Regulation (EC) 152/2009
Fatty acid composition (GC-FID, BF_3_)	ISO-12966-2/-4
Amino acid composition	ISO 13903:2005; Regulation (EC) 152/2009
Calorific value	ISO 1928:2020

## Data Availability

The data presented in this study are available on request from the corresponding author.

## Sample Availability

Samples of the insect protein hydrolysate is available from the authors
